# Ultrasonographic Assessment of Atherosclerotic Renal Artery Stenosis in Elderly Patients with Chronic Kidney Disease: An Italian Cohort Study

**DOI:** 10.3390/diagnostics12061454

**Published:** 2022-06-13

**Authors:** Yuri Battaglia, Fulvio Fiorini, Pietro Gisonni, Massimo Imbriaco, Paolo Lentini, Matthias Zeiler, Luigi Russo, Michele Prencipe, Domenico Russo

**Affiliations:** 1 Department of Medicine, University of Verona, 37129 Verona, Italy; 2 Nephrology and Dialysis Unit, Pederzoli Hospital, 37019 Peschiera del Garda, Italy; 3 Division of Nephrology and Dialysis, “Santa Maria della Misericordia” Hospital, 45100 Rovigo, Italy; fulvio.fiorini@aulss5.veneto.it; 4 Department of Radiology, University of Naples Federico II, 80100 Naples, Italy; pietro.gisonni@unina.it (P.G.); massimo.imbriaco@unina.it (M.I.); 5 Nephrology and Dialysis Unit, San Bassiano Hospital, 36061 Bassano del Grappa, Italy; paolo.lentini@yahoo.it; 6 Nephrology and Dialysis Unit, “Carlo Urbani” Hospital, 60035 Jesi, Italy; mrhz1@yahoo.com; 7 Nephrology and Dialysis Unit, “Ospedale del Mare”, 80100 Naples, Italy; luigirusso82@hotmail.it; 8 Division of Nephrology, Casa Sollievo della Sofferenza, 71100 San Giovanni Rotondo, Italy; mikprenc@libero.it; 9 Department of Public Health, University of Naples Federico II, 80100 Naples, Italy; domenicorusso51@hotmail.com

**Keywords:** atherosclerotic renal artery stenosis, chronic kidney disease, coronary artery calcification, elderly patients, duplex ultrasound

## Abstract

Although atherosclerotic renal artery stenosis (ARAS) is strictly associated with high cardiovascular risk and mortality, it often may remain unrecognized being clinically silent and frequently masked by co-morbidities especially in elderly patients with coexisting chronic kidney disease (CKD). The present observational study was conducted in elderly CKD-patients with atherosclerosis on other arterial beds. The aims were assessment of (1) ARAS prevalence; (2) best predictor(s) of ARAS, using duplex ultrasound; and (3) cardiovascular and renal outcomes at one-year follow-up. The cohort was represented by 607 consecutive in-patients. Inclusion criteria were age ≥65 years; CKD stages 2–5 not on dialysis; single or multiple atherosclerotic plaque on epiaortic vessels, abdominal aorta, aortic arch, coronary arteries, peripheral arteries that had been previously ascertained by one or more procedures. Duplex ultrasound was used to detect ARAS. Multiple regression analysis and ROS curve were performed to identify the predictors of ARAS. ARAS was found in 53 (44%) out of 120 patients who met the inclusion criteria. In univariate analysis, GFR (b = −0.021; *p* = 0.02); hemoglobin (b = −0.233; *p* = 0.02); BMI (b = 0.134; *p* = 0.036) and atherosclerosis of abdominal aorta and/or peripheral vessels (b = 1.025; *p* < 0.001) were associated with ARAS. In multivariable analysis, abdominal aorta and/or peripheral atherosclerosis was a significant (*p* = 0.002) predictor of ARAS. The area under the ROC curve was 0.655 (C.I. = 0.532–0.777; *p* = 0.019). ARAS is common in older CKD patients with extra-renal atherosclerosis, with the highest prevalence in those with aortic and peripheral atherosclerosis. ARAS may pass by unnoticed in everyday clinical practice.

## 1. Introduction

Atherosclerotic stenosis of renal artery (ARAS) is caused by the presence of atherosclerotic plaques at the proximal renal vessel or the ostium [1]. Over time the stenosis can worsen and often lead to a reduced blood flow of the renal cortex. The consequence of kidney ischemia is the activation of the renin-angiotensin-aldosterone system, resulting in renal and extrarenal hemodynamic effects, peripheral vasoconstriction, sodium/water retention, cortical hypoxia, local release of cytokine, and irreversible parenchymal injury [2]. Clinically, ARAS is responsible for poor control of hypertension, more frequent hospitalization, new onset of chronic kidney disease (CKD), faster progression of pre-existing CKD toward dialysis (ESRD) [3,4,5] and ultimately it increases cardiovascular risk and mortality [6,7]. Indeed, ARAS is independently associated with hypertension, heart failure, coronary artery disease, and peripheral artery disease [8,9].

ARAS remains underdiagnosed being entirely asymptomatic in mild/moderate stenosis [10] on one hand and frequently masked by the presence of atherosclerosis on other arterial beds on the other hand [11]. This is the case for elderly atherosclerotic patients with CKD [4]. Indeed, the data on the occurrence of ARAS are contrasting in this population [12,13,14], ranging from 5% up to 22% [15].

Although digital subtraction angiography represents the gold standard to ascertain ARAS, the procedure cannot be used for the screening program. It is invasive and potentially harmful due to the administration of contrast medium, especially in CKD patients [16].

On the other hand, duplex ultrasound (US) remains an accurate method of ARAS diagnosis (sensitivity 91–100% and sensibility 82–91%) and is useful for population screening. Indeed, it is considered the first-line examination in patients with suspected ARAS, according to the latest guidelines of the European Society of Cardiology [17]. Furthermore, it may be repeated for follow-up studies to evaluate the plaque progression and it can provide information for more invasive assessment. Because it is inexpensive, non-invasive, widely available, duplex ultrasound may be regarded as the gold-standard imaging technique to assess ARAS in everyday clinical practice, if performed by an expert operator [18].

The main aim of the present study was to assess the presence of ARAS, using duplex ultrasound, in an elderly CKD-patients cohort with ascertained atherosclerotic plaque on other arterial beds. As a secondary aim potential predictors or associations of ARAS with coronary artery calcification, biochemical data, and demographic characteristics were investigated. The final aim was to establish, the occurrence of cardiovascular and renal outcomes in patients with ARAS at one-year follow-up.

## 2. Methods

This retrospective, observational, longitudinal study was carried out on consecutive in-patients at the University of Naples. Inclusion criteria were: (1) age ≥ 65 years; (2) CKD stages 2 to 5 not on dialysis; (3) single or multiple atherosclerotic plaques on epi-aortic vessels, abdominal aorta, aortic arch, coronary arteries, peripheral arteries that had been previously ascertained by one or more procedures such as multislice computed tomography (MSCT) angiography, arteriography, MSCT-coronary, nuclear magnetic resonance, ultrasonography. Exclusion criteria were: (1) medical history of renal artery stenosis; (2) kidney transplant; (3) dialysis treatment. The procedures were in agreement with the Declaration of Helsinki and written informed consent was obtained from all the participants. The ethical approval was granted by the Ethics Committee for Human Research (Code 112, 2018).

Before US measurement, clinical examination, relevant data to personal and family medical history, and routine biochemistry were collected. The occurrence of fatal and no-fatal cardiovascular events, therapeutic treatments of ARAS or other endovascular plaques, and starting dialysis were recorded one year after the US measurement.

### 2.1. Ultrasound Technique

The presence of ARAS was assessed by duplex mode of ultrasound, namely B-mode and doppler, in all patients who met inclusion criteria. Duplex ultrasound was carried out according to the official recommendations of American and European ultrasound societies [19]. The procedure was performed with a 2 to 5 MHz curved array transducer with the patient fasted for 8 to 12 h before the examination to reduce attenuation of the ultrasound beam by air in the overlying bowel. The doppler sample volume covered the entire width of the renal artery trunk and an appropriate angle of insonation (<60°) was kept. The diagnostic criteria of significant renal artery stenosis (≥60%) were both an increased peak systolic velocity (≥200 cm/s) and an increased renal-to-aorta peak systolic velocity (≥3.5). If peak aortic velocity was less than 40 cm/s or greater than 100 cm/s due to decreased cardiac function or atherosclerosis, an absolute peak systolic velocity in the renal artery greater than 180 cm/s was used [20]. Degrees more than 60° of renal stenosis were taken into account, independently by the clinical relevance.

### 2.2. Other Data

Presence and extent of coronary artery calcification were assessed by multislice coronary computed tomography and scored in Agatston Unit, as described elsewhere [21]. Briefly, calcifications present in each coronary artery were scored and summed to obtain the total coronary calcium score (TCS).

Plaque on epi-aortic vessels, abdominal aorta, and peripheral arteries was defined as the presence of intima-medial thickness greater than 1.0 mm, according to the clinical practice standards [22].

Low-density lipoprotein cholesterol was calculated using the Friedewald equation. Hypertension was defined as blood pressure SBP > 140 mmHg and/or DBP > 90 mmHg. Renal function was assessed by 24/h measured creatinine clearance (GFR) and was staged according to KDOQI guidelines [23]. Dyslipidemia was defined according to ESC/EAS Guidelines for the management of dyslipidemias [24]. Intact parathyroid hormone was assayed by chemiluminescent immunometric method (Diagnostic Products, Los Angeles, CA, USA) and high-sensitivity C-reactive protein by the immunoturbidimetric method.

### 2.3. Statistical Analysis

Categorical variables are expressed as frequencies (percentage); and continuous variables as the median and interquartile range (IQR). Chi-square analysis and Kruskal-Wallis test were used for comparison between categorical or continuous variables, respectively.

All variables that resulted in significance (*p* < 0.05) at univariate analysis were reported in logistic regression analysis to measure their predictive role using ARAS as the outcome variable.

Receiver operating curve (ROC) was generated and area under the curve was calculated to assess the diagnostic accuracy of variable(s) for detecting ARAS. Combined sensitivities and specificities were visualized in the ROC curve. AUC of 0.5–0.7 indicates low accuracy, 0.7–0.9 indicates moderate accuracy and 0.9–1.0 indicates high accuracy. A two-sided *p*-value of <0.05 indicated statistical significance.

Statistical analysis was performed using SPSS statistical software (version 28, IBM Corp. Armonk, NY, USA).

## 3. Results

607 consecutive in-patients were screened from January 2015 to December 2018 and data of 120 patients who met inclusion criteria were collected before and after US evaluation. CKD stages were: II = 21%; III = 36.1%; IV = 26.1%; V = 16.8%. The main cause of CKD was unknown (51%), followed by biopsy-proven diabetic nephropathy (15%), glomerulonephritis (12%); and miscellanea (22%), namely Autosomal Dominant Polycystic Kidney Disease, interstitial nephritis, and kidney stones.

All patients were in treatment with antihypertensive medications, including calcium channel blockers (60%), angiotensin-converting enzyme inhibitors (ACE-I) (85%), angiotensin II receptor blocker (ARB, 81%), combination of ACE-I and ARB (76%).

ARAS was ascertained in 53 cases (44%) (ARAS-patients); renal artery stenosis was found on right renal artery in 58% of cases; ARAS was bilateral in one case; remaining 67 subjects were regarded as controls. Median percentage of stenosis was 70% (IQR: 55–75%).

There was no significant difference in the number of antihypertensive medications and statins between ARAS patients and controls. More than half of total cohort (67%) was in treatment with statins alone or in combination with ezetimibe.

Family history of cardiovascular events occurred in 22 ARAS patients and 27 controls. Clinical characteristics and blood chemistry of patients are reported in Table 1 and Table 2.

Significant differences were found in basal BMI, GFR, hemoglobin, pulse pressure, serum calcium concentration, and TCS between ARAS patients and controls. There was no difference in markers of mineral metabolism (intact parathyroid hormone, serum calcium, and phosphorus), dyslipidemia (total cholesterol, triglycerides, high-density lipoprotein cholesterol, and low-density lipoprotein cholesterol), inflammation (homocysteine, fibrinogen, and high-sensitivity C-reactive protein), nutrition (total protein and serum albumin) and therapy (antiplatelet agents and statins). No difference was found in blood pressure levels between ARAS patients and controls.

In univariate analysis GFR (b = −0.021; *p* = 0.02), hemoglobin (b = −0.233; *p* = 0.02); BMI (b = 0.134; *p* = 0.036) and presence of plaque on abdominal aorta and/or peripheral vessels (b = 1.025; *p* < 0.001) were associated with ARAS. Results of multivariable logistic analysis are reported in Table 3.

**Figure 1:**
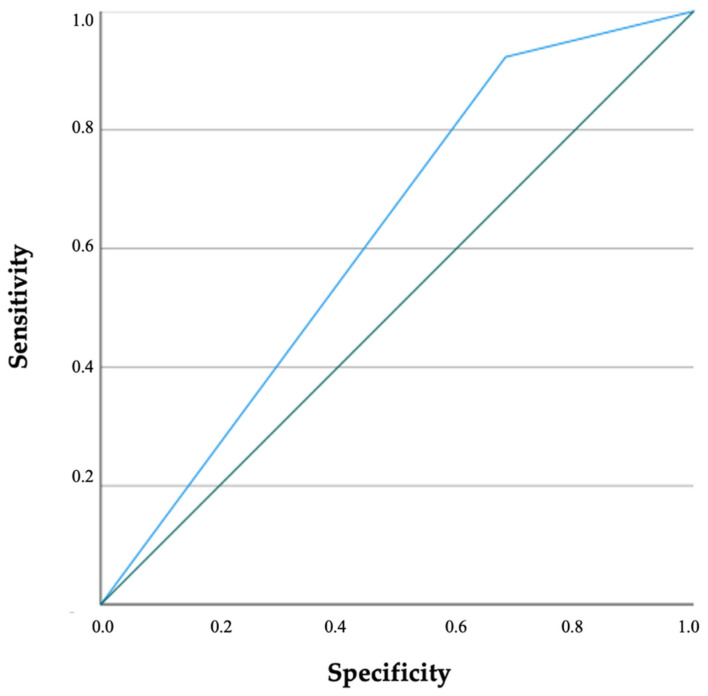
Receiver operating characteristic analysis of presence of abdominal aorta and/or peripheral vessels atherosclerosis on ARAS is illustrated. ARAS: Atherosclerotic stenosis of the renal artery.

**Figure 2:**
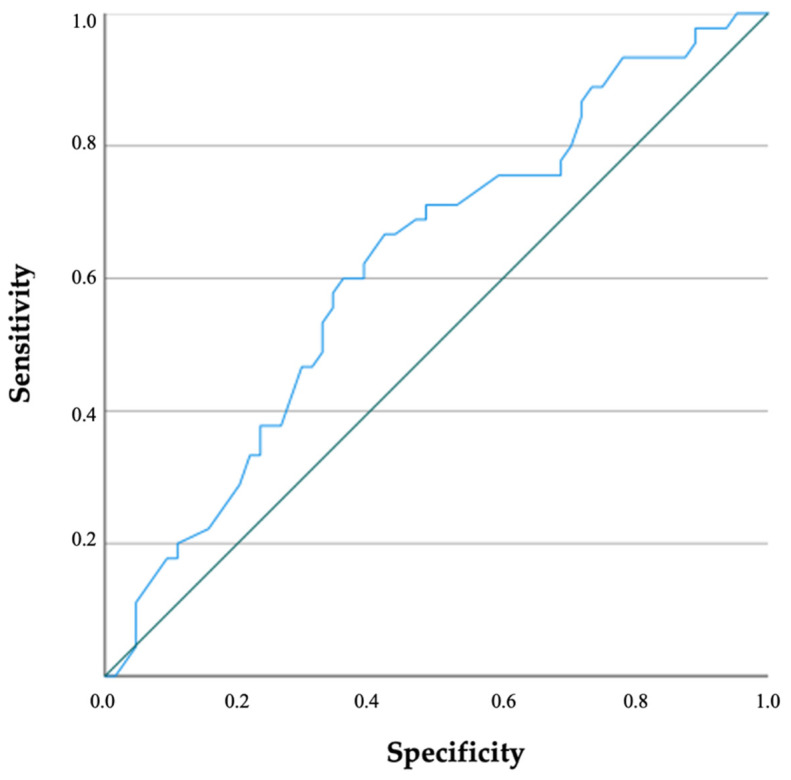
Receiver operating characteristic analysis of glomerular filtration rate on ARAS is illustrated. ARAS: Atherosclerotic stenosis of renal artery.

The presence of plaque on the abdominal aorta and/or peripheral arteries and GFR were the unique significant predictors of ARAS. Generated ROC curve by presence of plaque on abdominal aorta and/or on peripheral vessels and GFR are shown in Figure 1 and Figure 2; and the area under the curve was 0.655 (C.I. = 0.532–0.777; *p* = 0.019) and 0.623 (C.I. = 0.517–0.729; *p* = 0.030), respectively.

Evaluating the clinical files of patients one year after the study closing, it was found that no ARAS-patients had been treated with angioplasty, five patients had initiated dialysis (vs. n.1 of controls; *p* = ns), two patients had experienced no fatal cardiovascular event (vs. n.0 of controls; *p* = ns), eight patients (vs. n.3 of controls; *p* = ns) had undergone cardiac revascularization.

## 4. Discussion

In this study, we recorded a high prevalence of ARAS in elderly CKD patients with diagnosed atherosclerotic plaque on other arterial districts. ARAS was found in 44% of the study population, indicating that almost a half of elderly atherosclerotic patients with CKD are likely to have ARAS. With respect to this finding, it is worth noting that the overall occurrence of ARAS observed in our study was higher than that reported in hypertensive patients, in patients with suspected and proven (coronary) heart disease, and in patients undergoing aortic or lower-extremity angiography, ranging from 5 to 25% [25].

Furthermore, ARAS could be even more frequent by adopting less restricted inclusion criteria than those of the present study such as the assessment of ARAS in younger CKD patients with atherosclerosis in other arterial districts [26,27].

Notably, our patients were unaware of the presence of ARAS before doppler ultrasound were performed. This finding suggests that the occurrence of ARAS may pass unnoticed in the setting of everyday clinical practice being associated with masking comorbidities in many cases [6,28,29]. Indeed, ARAS is often underdiagnosed as outlined by a large study population that evaluated symptomatic patients of the United States Medicare [30] and in a community-based screening study among free-living adults of more than 65 years of age [10].

Therefore, the rate of occurrence of ARAS seems dependent on the characteristics of the study population and on selection criteria [8,31].

A further analysis of our study showed that ARAS was predicted by atherosclerotic plaques on the abdominal aorta and/or on peripheral vessels, but not by variables of mineral metabolism, dyslipidemia, inflammation, nutrition, and blood pressure. These findings are in line with previous studies [32,33], where atherosclerosis of the aorta and/or peripheral artery was strictly correlated with ARAS. In a retrospective observational study [34], calcification on the abdominal aorta predicted the presence of renal artery stenosis in adult patients. Although a large population was investigated, no data regarding the kidney function of enrolled patients was reported. In addition, aortic calcification was assessed with a 64-slice multidetector computed tomography scanner, an expensive and time-consuming procedure.

Interesting results emerged when the renal event occurrence was recorded at one-year follow-up after duplex doppler ultrasound, the inception to dialysis was more frequent in ARAS patients. Although not statistically significant, these data could suggest that ARAS might be a potential cause of a faster decline of kidney function in the elderly population. These results are consistent with other studies reporting that ARAS was responsible for the progressive loss of renal mass and function over time in 21 to 27% of patients [35,36,37]. Furthermore, in 9.2% to 14% of patients new to dialysis, ARAS was regarded as the main cause of end-stage renal disease [6]; and occurrence of ARAS was found in 40.8% of patients upon initiation of dialysis [7]. Nonetheless, it is worth noting that the role of ARAS, as a causative factor of either new onset of CKD or faster decline of pre-existing CKD, is still questioned. Indeed, in patients with renal artery stenosis, significant progression towards end-stage renal disease is rare or absent. In addition, patients with ARAS did not show a clinical picture different from other patients with signs of atherosclerosis elsewhere [38,39,40,41,42].

Interestingly, when examining the hypertensive status, ARAS patients did not show higher blood pressure than controls and there was no significant difference in the number of antihypertensive medications between ARAS patients and controls. This result seems to indicate that the degree of hypertension is variable in ARAS patients, although an intensive control of blood pressure is mandatory.

Regarding coronary TCS in ARAS patients, our study demonstrated that the median TCS (399 IQR 60–400 AU) was two times higher than controls (190 IQR 130–607 AU) and four times than the threshold risk for cardiovascular events in CKD patients not on dialysis (which is positioned at the level of 100 AU) [43,44,45,46,47]. Moreover, at one-year follow-up after duplex ultrasound, cardiac revascularization was required more frequently in ARAS patients.

This study has strengths and limitations. Our results provide evidence of atherosclerotic renal artery stenosis having a high prevalence in elderly patients with CKD and atherosclerotic lesions on the abdominal aorta and/or on peripheral vessels.

Some limitations of our study should be mentioned. Firstly, patients were evaluated only by ultrasonography. Although results of ultrasonography are operator dependent and limited by the influence of bowel gas patterns as well as by body fat composition, low rates of technical failure are observed in experienced hands [48]. Another limitation is the short follow-up period and the absence of clinical and biochemical data at one year follow-up after duplex ultrasound. Multicenter prospective studies on elderly CKD should be considered to provide more robust results since few studies on silent ARAS have been conducted in elderly CKD patients, not on dialysis. Finally, we did not collect any data regarding the voluptuary habits (smoking, alcohol consumption, and coffee drinks) [49] and the physical activity that could affect the atherosclerotic lesions [10,50,51].

In conclusion, our study demonstrates that ARAS is common in elderly patients with extra-renal atherosclerosis, with the highest prevalence in those with aortic and peripheral atherosclerotic artery disease. Routinary use of duplex ultrasound should be encouraged to unmasked silent atherosclerotic lesions on renal artery in the clinical practice. Further prospective and multicenter studies should be performed to investigate the real burden of ARAS in elderly CKD patients.

## Figures and Tables

**Table 1:** Clinical characteristics of patients as whole cohort and as classified group according to presence of renal artery stenosis.

	Whole Cohort n. 120	ARAS n. 53 (44%)	NO ARAS n. 67 (56%)	*p*-Value
Male/Female, n. (%)	82/38 (68.3/31.7)	38/15 (71.7/28.3)	44/24 (65.6/34.3)	0.48
Age, years *	73 (70–79)	74 (70–79)	73 (69–79)	0.12
BMI, Kg/m^2^ *	26.1 (24.1–30.0)	24.9 (22.5–28.3)	26.9 (25.5–31.5)	0.01
CKD vintage, months *	36 (24–84)	42 (24–93)	36 (24–72)	0.70
Hypertension, n. (%)	120 (100)	53 (100)	67 (100)	
Hypertension vintage, months *	120 (48–222)	132 (48–240)	96 (57–192)	0.31
Diabetes, n. (%)	36 (30.0)	14 (26.4)	22 (32.8)	0.09
Diabetes vintage, months *	120 (72–240)	126 (63–240)	120 (72–240)	0.82
Dyslipidemia, n. (%)	81 (67.5)	39 (73.6)	42 (62.7)	0.75
Past CV events, n. (%)	44 (36.7)	24 (45.3)	20 (30.0)	0.41
Plaque on epiaortic vessels, n. (%)	96 (80.0)	37 (69.8)	59 (93.7)	0.12
Plaque on abdominal aorta/ peripheral arteries, n. (%)	60 (50.0)	36 (67.9)	24 (35.8)	0.01
Plaque on coronaries, n. (%)	34 (28.0)	15 (28.0)	19 (28.0)	0.85
TCS, Agatston Unit *	190 (60–400)	399 (130–607)	176 (50–342)	0.03

* Data are expressed as Median (IQR).

**Table 2:** Blood pressure levels and biochemistry of patients as whole cohort and as classified group according to presence of renal artery stenosis.

	Whole Cohort n. 120	ARAS n. 53 (44%)	NO ARAS n. 67 (56%)	*p*-Value
Systolic Blood Pressure, mmHg *	130 (120–140)	140 (130–150)	130 (120–140)	0.10
Diastolic Blood Pressure, mmHg *	80 (70–80)	80 (70–80)	80 (70–80)	0.33
Mean Blood Pressure, mmHg *	96 (90–105)	96 (90–104)	96 (90–104)	0.94
Pulse Pressure, mmHg *	60 (50–70)	60 (50–70)	50 (40–60)	0.01
GFR, mL/s *	0.67 (0.35–0.98)	0.52 (0.33–0.90)	0.82 (0.42–1.07)	0.03
Total cholesterol, mmol/L *	4.58 (3.83–5.30)	4.42 (3.65–5.30)	4.73 (4.03–5.30)	0.09
Triglycerides, mmol/L *	1.40 (1.02–1.89)	1.35 (0.97–1.76)	1.46 (1.06–2.05)	0.37
HDL-Cholesterol, mmol/L *	1.16 (0.91–1.53)	1.06 (0.93–1.27)	1.22 (0.91–1.55)	0.25
LDL-Cholesterol, mg/dL *	104 (82–129)	95 (74–131)	109 (85–129)	0.22
Homocysteine, μmol/L *	20 (18–25)	21 (16.6–26.9)	20 (17–23.8)	0.59
C-Reactive Protein, mg/dL *	0.33 (0.32–0.95)	0.33 (0.32–1.26)	0.33 (0.32–0.74)	0.50
Fibrinogen, μmol/L *	12.0 (9.9–15.2)	11.8 (10.0–15.2)	12.4 (9.9–15.3)	0.91
Haemoglobin, g/L *	127 (108–138)	119 (103–136)	129 (111–141)	0.02
Serum Proteins, g/L *	69 (63–74)	68 (60–74)	69 (65–74)	0.47
Serum Albumin, g/dL *	42 (37–46)	41 (35–46)	42 (37–46)	0.41
Serum Phosphorus, mmol/L *	1.13 (0.97–1.36)	1.16 (0.90–1.36)	1.13 (1.0–1.36)	0.44
Serum Calcium, mmol/L *	4.65 (4.50–4.85)	4.55 (4.40–4.80)	4.70 (4.50–4.85)	<0.01
PTH, ng/L *	76 (50–130)	93 (56–141)	70 (46–114)	0.69
Uric Acid, μmol/L *	387 (315–452)	393 (315–458)	387 (303–434)	0.58

* Data are expressed as Median (IQR).

**Table 3:** Results of multivariate analysis.

	B	S.E.	Wald	gl	*p*-Value	Exp(B)	95.0% CI for Exp(B) LB	95.0% CI for Exp(B) UB
Plaque on AA/PV	1.311	0.413	10.065	1	0.002 *	3.711	1.651	8.345
GFR, mL/min	−0.042	0.021	3.868	1	0.049 *	0.959	0.920	1.000
Haemoglobin, g/dL	−0.002	0.252	0.000	1	0.994	0.998	0.609	1.636
BMI, kg/m^2^	−0.114	0.079	2.061	1	0.151	0.892	0.764	1.042

Dependent Variable: ARAS. AA: Abdominal Aorta; BMI: Body Mass Index; GFR: glomerular filtration rate (as 24 h measured creatinine clearance); PV: peripheral Vessels. * Statistically significant.

## Data Availability

Not available.
